# Impact of Facility Volume on Therapy and Survival for Endometrial Cancer: A Retrospective Multicenter Study

**DOI:** 10.3390/cancers18071050

**Published:** 2026-03-24

**Authors:** Vincenzo Dario Mandato, Anna Myriam Perrone, Debora Pirillo, Gino Ciarlini, Gianluca Annunziata, Alessandro Arena, Carlo Alboni, Ilaria Di Monte, Vito Andrea Capozzi, Andrea Amadori, Ruby Martinello, Federica Rosati, Marco Stefanetti, Andrea Palicelli, Giacomo Santandrea, Renato Seracchioli, Roberto Berretta, Lorenzo Aguzzoli, Federica Torricelli, Pierandrea De Iaco

**Affiliations:** 1 Unit of Obstetrics and Gynecologic Oncology, Azienda USL-IRCCS di Reggio Emilia, 42123 Reggio Emilia, Italy; debora.pirillo@ausl.re.it (D.P.); gino.ciarlini@ausl.re.it (G.C.); gianluca.annunziata@ausl.re.it (G.A.); lorenzo.aguzzoli@ausl.re.it (L.A.); 2 Division of Oncologic Gynecology, IRCCS Azienda Ospedaliero–Universitaria di Bologna, 40138 Bologna, Italy; myriam.perrone@aosp.bo.it (A.M.P.); pierandrea.deiaco@unibo.it (P.D.I.); 3 Department of Medical and Surgical Sciences, University of Bologna, 40138 Bologna, Italy; alessandro.arena@unibo.it (A.A.); renato.seracchioli@unibo.it (R.S.); 4 Division of Gynaecology and Human Reproduction Physiopathology, IRCCS Azienda Ospedaliero–Universitaria di Bologna, 40138 Bologna, Italy; 5 Department of Medical and Surgical Sciences for Mother, Child and Adult, University of Modena and Reggio Emilia, 41124 Modena, Italy; carlo.alboni@aou.mo.it (C.A.); ilaria.dimonte@aou.mo.it (I.D.M.); 6 Department of Obstetrics and Gynecology, University of Parma, 43126 Parma, Italy; vitoandrea.capozzi@ao.pr.it (V.A.C.); roberto.berretta@unipr.it (R.B.); 7 Gynecology Unit, Ospedale di Forlì, 47121 Forlì, Italy; dottamadori@gmail.com; 8 Section of Obstetrics and Gynecology, Department of Medical Sciences, University of Ferrara, 44124 Ferrara, Italy; ruby.martinello@unife.it; 9 Gynecological and Obstetrical Unit, Infermi Hospital, 47923 Rimini, Italy; federica.rosati@auslromagna.it (F.R.); marco.stefanetti@auslromagna.it (M.S.); 10 Pathology Unit, Azienda USL-IRCCS di Reggio Emilia, 42123 Reggio Emilia, Italy; andrea.palicelli@ausl.re.it (A.P.); giacomo.santandrea@ausl.re.it (G.S.); 11 Laboratory of Translational Research, Azienda USL-IRCCS di Reggio Emilia, 42123 Reggio Emilia, Italy; federica.torricelli@ausl.re.it

**Keywords:** endometrial cancer, volume center, survival, centralization, laparoscopy

## Abstract

**Background**: Endometrial cancer (EC) is the most common gynecological malignancy in Western countries. Although international guidelines recommend that patients with EC be treated at specialized oncology centers, many are still managed by general gynecologists. This study aimed to evaluate the influence of facility volume on treatment strategies and survival outcomes among EC patients. **Methods**: This is a retrospective multicenter study comparing 971 patients with EC treated at medium-volume centers (CVMs) (11–29 cases/year) with 1431 patients treated at high-volume centers (CVAs) (≥30 cases/year). Patient characteristics were recorded, including age, body mass index (BMI), American Society of Anesthesiologists (ASA) score, comorbidities, surgical approach, lymphadenectomy, total number of lymph nodes removed, number of positive lymph nodes, length of hospital stay, histological characteristics, ESMO-ESGO (European Society for Medical Oncology–European Society of Gynaecological Oncology) classification system, adjuvant treatment, recurrence, progression-free survival (PFS), and overall survival (OS). Postoperative fever, hemoglobin changes, and blood transfusions were also reported. **Results**: Compared with patients treated at the MVCs, patients treated at the HVCs were younger (mean age, 65 vs. 66.4 years, *p* = 0.03) and had a lower rate of comorbidities (41% vs. 55%, *p* < 0.001). Patients treated at HVCs were mostly in higher ESMO-ESGO recurrence risk classes (*p* < 0.001), were treated mostly laparoscopically (58% vs. 47%, *p* < 0.001) and had better staging (higher number of lymph nodes harvested (mean 19 vs. 11, *p* < 0.001) and more peritoneal biopsies performed (27% vs. 14%, *p* < 0.001). HVC patients had fewer complications and received less adjuvant therapy (40% vs. 50%, *p* < 0.001) but, when treated, received chemotherapy more frequently, showed mostly loco-regional recurrences (34% vs. 14%) and fewer extra-abdominal recurrences (34% vs. 54%). HVC patients had better PFS and OS. Center volume was found to be an independent factor influencing PFS in multivariate analysis. **Conclusions**: All EC patients should be centrally managed to receive superior treatment to improve postoperative recovery and oncological outcomes, particularly for patients with more-aggressive tumors.

## Simple Summary

Endometrial cancer (EC) is the most common gynecological malignancy in Western countries, with a rising incidence in nations undergoing rapid Westernization. Although it is generally considered straightforward to manage and is often treated by general gynecologists, this approach may not be optimal for all cases. In this study, based on real-world data, we found that centralizing care in high-volume specialized centers (HVCs) significantly impacts the quality of treatment. Specifically, centralization improves oncological outcomes for patients with tumors that have a higher risk of recurrence. Identifying a universal threshold for high-volume centers remains a challenge, as the number of annual surgeries used to define “high volume” differs globally.

## 1. Introduction

Endometrial cancer (EC) is the most common malignancy of the female genital tract in Western and emerging countries [1]. Its incidence has increased worldwide, also in young women [2]. It is the only cancer with reduced survival over the past four decades [3]; despite EC mortality decreasing at the global level, mortality has increased in many countries [4]. It is generally considered easy to treat and is often managed even by general gynecologists. Treatment in low-volume hospitals (<10 cases per year) was associated with differences and discrepancies in clinical management [5]. The use of sentinel lymph node biopsy has also fostered this practice. However, if mistreated, EC has a high lethality due to its poor response to chemotherapy in the cases of advanced stage and recurrence [6]. Recently, the integration of molecular profiling has significantly stratified the management of endometrial cancer (EC). Consequently, the implementation of a Molecular Tumor Board (MTB) has become essential to guide personalized therapeutic strategies and ensure optimal patient care [7]. Although there is strong evidence for the need to centralize ovarian cancer [8], the impact of centralization and high-volume centers on EC treatment is not yet clarified [9]. The number of patients to be treated each year to define a high-volume center is also not yet well defined. The number varies depending on the year of publication of the studies and the population under examination [10,11,12,13,14,15]. The early literature defined high-volume centers as those handling ≥ 10 cases annually; however, contemporary data have shifted this benchmark to ≥80 cases per year [11]. The Italian Ministry of Health defines reference centers as facilities managing at least 20 cases annually. In this context, the Emilia-Romagna region demonstrates high centralization for both ovarian and endometrial cancer (EC); specifically, 94% of EC patients are treated in hospitals meeting this minimum volume threshold [16]. A high-volume center should ensure both the diagnostic phase and the treatment of patients with endometrial cancer. Accurate preoperative diagnosis could enable the identification of low-risk endometrial cancer cases that may be managed in lower-volume centers [9]. Preoperative staging relies on expert-led transvaginal or transrectal ultrasonography (US) and pelvic Magnetic Resonance Imaging (MRI) to evaluate myometrial invasion, cervical stromal involvement, and adnexal status. Furthermore, chest–abdominal–pelvic Computed Tomography (CT) or Positron Emission Tomography (PET) scans are recommended to exclude nodal metastases, peritoneal carcinomatosis, and distant secondary lesions [6]. Currently, US and MRI are the primary modalities employed for the preoperative assessment of myometrial invasion depth. While both techniques demonstrate comparable diagnostic efficacy, US remains the preferred first-line approach due to its cost-effectiveness and shorter acquisition times [9,10,17,18]. Nevertheless, the accuracy of US-based preoperative staging can be significantly compromised in complex clinical scenarios. For instance, the presence of concomitant uterine pathologies, such as leiomyomas or adenomyosis, can obscure the tumor–myometrial interface [19]. Notably, in low-risk EC patients, the specificity of MRI is significantly superior [20]. Furthermore, unlike the standardized protocols of MRI, US is inherently operator-dependent; its diagnostic reliability is highly contingent on the clinician’s expertise, which may lead to substantial inter-observer variability in staging results. Today, the integration of the US with radiomic models has made it possible to standardize subjective evaluations, overcoming critical issues and significantly improving accuracy in identifying myometrial infiltration [21].

In this study, we evaluated the impact of hospital volume on treatment patterns and clinical outcomes for EC patients.

## 2. Methods

In accordance with the journal’s guidelines, we will provide our data for independent analysis by a team selected by the Editorial Team for the purposes of additional data analysis or for the reproducibility of this study in other centers if such is requested.

### 2.1. Study Design

We analyzed the population described in our previous study in which details on recruitment, collected data and general characteristics of the population have been reported [22]. This multicenter study included all patients who underwent hysterectomy for endometrial cancer between 2000 and 2019 across seven hospitals in the Emilia-Romagna region of Northern Italy: the University Hospitals of Bologna, Modena and Reggio Emilia, Parma, and Ferrara, as well as the USL-IRCCS of Reggio Emilia, Ospedale di Forlì, and Ospedale degli Infermi (Rimini) [22].

Due to the considerable variability in thresholds used to define “high volume” facilities, in contrast to a broader consensus on “low volume” definitions in the literature, we established our classification based on a synthesis of international data. Specifically, low-volume hospitals were defined as those treating up to 10 cases per year [10,14]. To define medium-volume hospitals, we harmonized European [13,15,23] and non-European studies [12,14,24], the latter often using higher thresholds. Consequently, we defined medium-volume centers (MVCs) as those treating 11 to 29 cases/year and high-volume centers (HVCs) as those treating ≥ 30 cases/year.

### 2.2. Data Collection

Patients’ characteristics, including age, body mass index (BMI), American Society of Anaesthesiologists (ASA) classification system score, comorbidities such as diabetes and hypertension, were recorded. Vaginal hysterectomy, laparoscopy (LPS), laparotomy (LPT), peritoneal biopsy, peritoneal washing, pelvic lymph node dissection (PLND), paraaortic lymph node dissection (PALD), sentinel lymph node dissection (SLD), total lymph node retrieved, number of positive lymph nodes, duration of surgery, hospital length of stay, International Federation of Obstetrics and Gynaecology (FIGO) stage, histology, grade, lymphovascular space invasion (LVSI), European Society For Medical Oncology (ESMO)–European Society of Gynaecological Oncology (ESGO) class of risk [6], adjuvant treatment, recurrence, site of recurrence, death, total survival, progression-free survival (PFS), and overall survival (OS) were reported. Complications such as postoperative fever, hemoglobin variation, and a requirement for blood transfusions were also reported (Table 1).

### 2.3. Statistical Analysis

All statistical analyses were conducted using R software version 4.3.1 (R Foundation for Statistical Computing, Vienna, Austria). Analysis of association was performed by applying Fisher’s exact test for categorical variables and the ANOVA test for the comparison of continuous variables between two groups. OS was calculated as the period spent from the treatment surgery date to the date of death; patients who were alive at the end of the study were censored at the date of their last clinical follow-up.

PFS was calculated as the time from the date of surgery to the date of first documented recurrence. Patients who died without evidence of disease progression were censored at the date of death, and those alive without recurrence were censored at the date of their last clinical follow-up. Survival analyses were represented by Kaplan–Meier curves using the R “Survminer” package, and statistical differences were evaluated by the log-rank test. Significant statements refer to *p*-values lower than 0.05.

## 3. Results

### 3.1. Clinical Characteristics

In this study we included 2402 EC patients treated from 2000 to 2019 in eight Emilia Romagna clinical centers. Four HVCs and four MVCs were compared. In total, 1431 patients were treated in HVCs and 971 in MVCs (Table 1).

**Figure 1:**
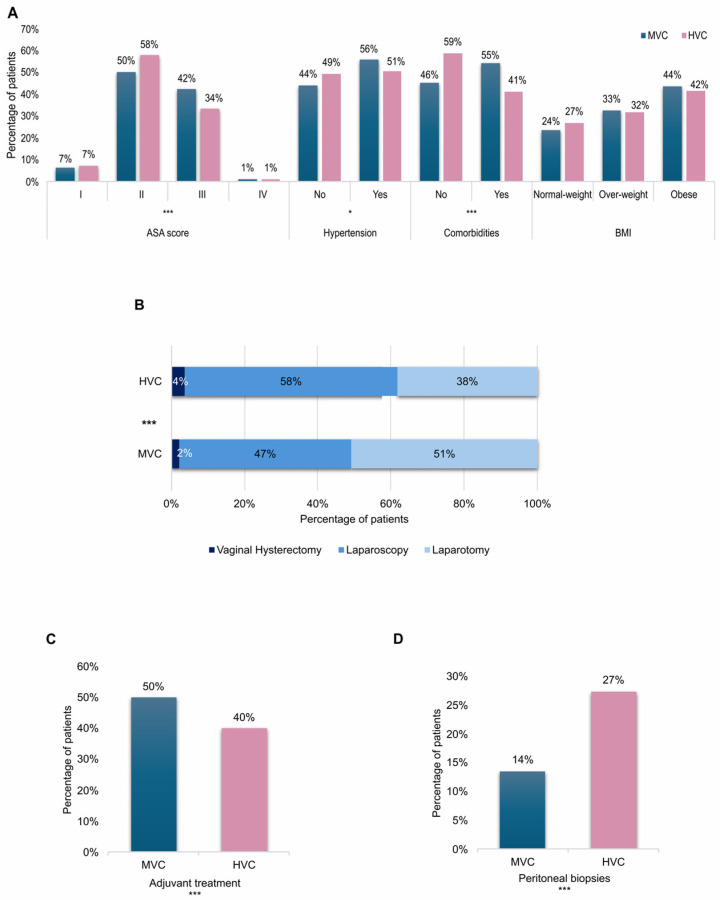
(**A**) Histograms showing the clinical characteristics of endometrial cancer patients subdivided between medium-volume centers and high-volume centers. (**B**) Stacked bar plot showing the distribution of different surgical approaches adopted in medium-volume centers and high-volume centers. (**C**) Histograms showing the percentage of patients who received adjuvant treatment in medium-volume centers and high-volume centers. (**D**) Histograms showing the percentage of patients who underwent peritoneal biopsies in medium-volume centers and high-volume centers. (* *p* < 0.05, *** *p* < 0.001).

From an initial analysis on clinical characteristics, it emerged that patients treated in HVCs were slightly younger than patients treated in MVCs (mean age 65 vs. 66.4, *p* = 0.03) and presented a better clinical status with a higher ASA score II percentage (58% vs. 50%) in spite of a lower ASA score III percentage (34% vs. 42%) (*p* < 0.001), milder cases of hypertension (51% vs. 56%, *p* = 0.016) and in general a lower percentage of comorbidities (41% vs. 55%, *p* < 0.001). No differences in terms of BMI were observed (Figure 1A). In HVCs a significantly higher percentage of patients was surgically treated with LPS (58% vs. 47%, *p* < 0.001) (Figure 1B) with a consequent reduction of bleeding (mean hemoglobin variation −1.6 vs. −1.8, *p* < 0.001), surgical times (mean 145 min vs. 177 min, *p* < 0.001) and days of hospitalization (mean 5.7 vs. 6.5, *p* < 0.001); moreover only 40% of patients treated in HVCs received adjuvant therapy, versus 50% of those treated in MVCs (Figure 1C). In HVCs, patients received a peritoneal biopsy in 27% of cases versus 14% in MVCs (*p* < 0.001) (Figure 1D), and even though no significant differences in the choice to perform PLNS or PALD were observed, a significantly higher number of collected lymph nodes (mean 21.5 vs. 19, *p* < 0.001) and positive lymph nodes (4% vs. 2%, *p* = 0.024) was registered in HVCs.

### 3.2. ESMO-ESGO Risk of Recurrence Stratification

**Figure 2:**
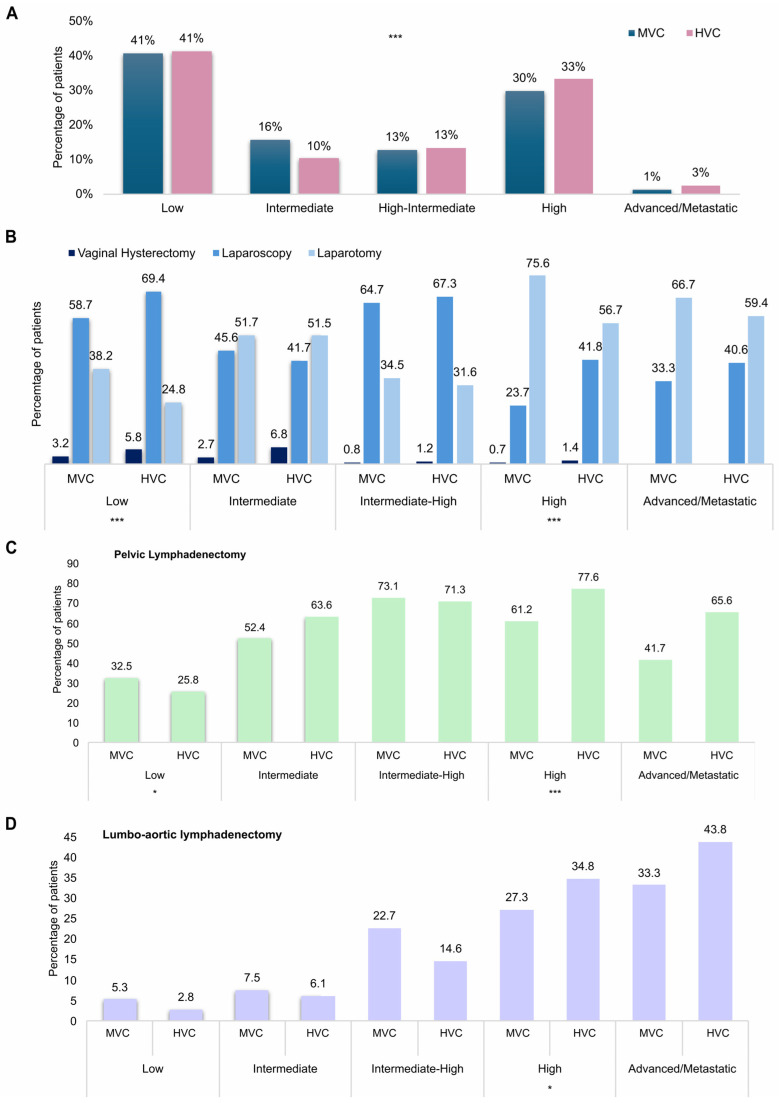
(**A**) Histograms showing the distribution of ESMO-ESGO risk in medium-volume centers and high-volume centers. (**B**) Histograms illustrating the surgical approach adopted in medium-volume centers and high-volume centers, stratified by ESMO-ESGO risk classification. (**C**) Histograms illustrating the percentage of patients who underwent pelvic lymphadenectomy in medium-volume centers and high-volume centers, stratified by ESMO-ESGO risk classification. (**D**) Histograms illustrating the percentage of patients who underwent lumbo-aortic lymphoadenectomy in medium-volume centers and high-volume centers, stratified by ESMO-ESGO risk classification. (* *p* < 0.05, *** *p* < 0.001).

To better understand the reasons of these differences we analyzed the two groups subdividing patients based on the risk of recurrence (ESMO-ESGO risk of recurrence stratification) 6. In particular, the HVC patient cohort was composed by a lower percentage of intermediate-risk patients (10% vs. 16%) and by a higher percentage of high-risk (33% vs. 30%) and advanced/metastatic patients (3% vs. 1%) (*p* < 0.001) (Figure 2A). Among high-risk patients, 60.5% of those treated in HVCs had a non-endometrioid histotype versus only 30% of those treated in MVCs (*p* < 0.001). Approximately 38% of low-risk patients managed at MVCs underwent LPT, compared with only 24.8% of those treated at HVCs. Conversely, LPS was performed in approximately 59% of low-risk patients at MVCs and in nearly 70% at HVCs (*p* < 0.001). Similarly, LPS was preferred in 41.8% of high-risk patients in HVCs versus only 23.7% of those in MVCs (*p* < 0.001) (Figure 2B). Likewise, the decision to perform lymphadenectomy differed between centers, particularly in low- and high-risk patients. Pelvic lymph node dissection was performed in 32.5% of low-risk patients in MVCs versus about 26% of low-risk patients in HVCs (*p* = 0.031). Consistently, high-risk patients received PLD in 77.6% of cases in HVCs versus 61.2% in MVCs (*p* < 0.001) and PALD in about 35% of cases in HVCs versus 27.3% in MVCs (*p* = 0.039) (Figure 2C,D).

### 3.3. Adjuvant Treatment

**Figure 3:**
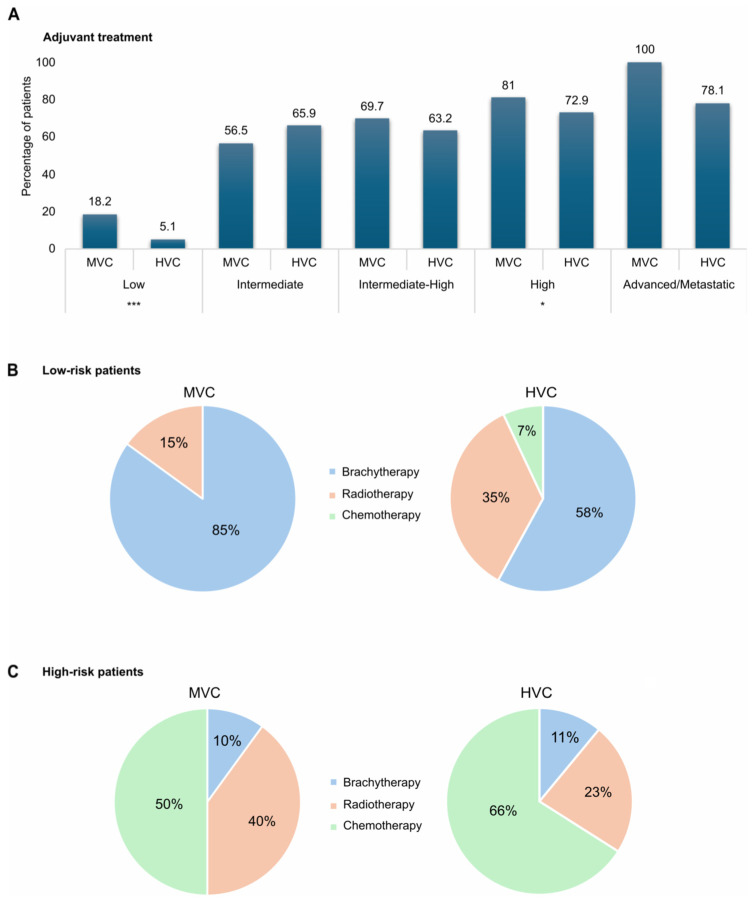
(**A**) Histograms illustrating the percentage of patients who received adjuvant treatment in medium-volume centers and high-volume centers, stratified by ESMO-ESGO risk classification. (**B**) Pie charts showing the distribution of adjuvant treatment types between medium-volume centers and high-volume centers in the subgroup of low-risk patients. (**C**) Pie charts showing the distribution of adjuvant treatment types between medium-volume centers and high-volume centers in the subgroup of high-risk patients (* *p* < 0.05, *** *p* < 0.001).

The choice to administer adjuvant therapy also differed between HVCs and MVCs for low- risk and high-risk patients. Overall, 18.2% of low-risk women were treated with adjuvant therapy in MVCs versus only 5.2% in HVCs (*p* < 0.001), while high-risk patients received adjuvant treatment in 81% of cases in MVCs vs. 72.9% in HVCs (*p* = 0.015) (Figure 3A). Interestingly in MVCs, low-risk patients who received adjuvant therapy were treated with brachytherapy alone in about 85% of cases and with radiotherapy (alone or in combination with brachytherapy) in 15% of cases, while in HVCs only 58% of low-risk patients received brachytherapy, 35% received radiotherapy and 7% received chemotherapy (*p* = 0.005) (Figure 3B). Along the same lines, high-risk patients treated with adjuvant therapy in MVCs received radiotherapy in a significantly higher percentage of cases than in HVCs (40% vs. 23%) despite having a lower percentage of chemotherapy (50% versus 66%) (*p* < 0.001) (Figure 3C).

### 3.4. Survival

**Figure 4:**
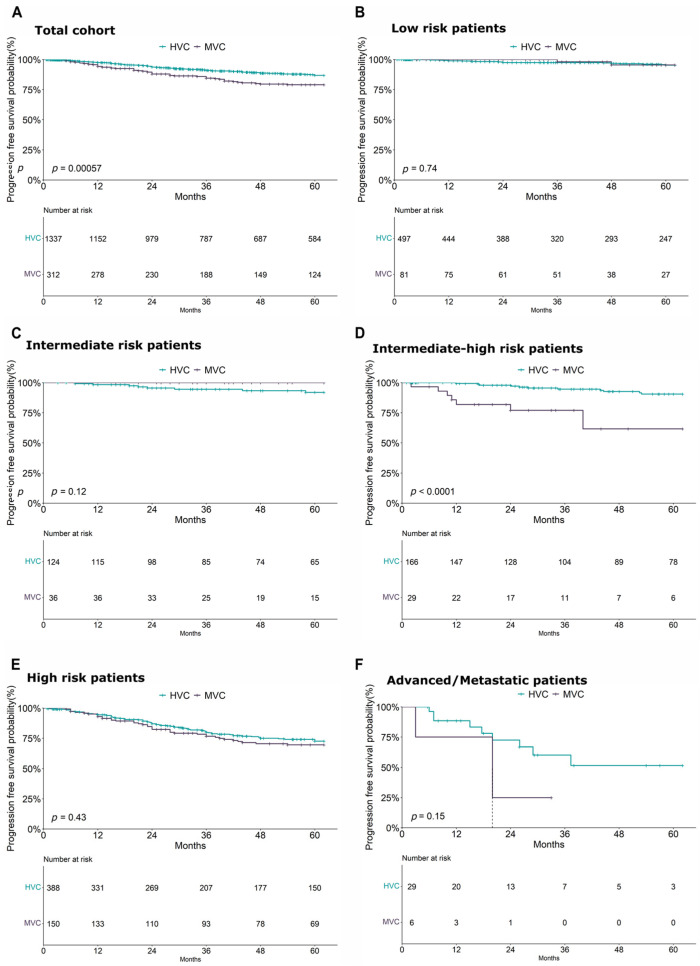
Kaplan–Meier curves illustrating the effect of center volume on progression-free survival in: (**A**) the entire patient cohort, (**B**) the sub-cohort of ESMO-ESGO low risk patients, (**C**) the sub-cohort of ESMO-ESGO intermediate risk patients, (**D**) the sub-cohort of ESMO-ESGO intermediate–high risk patients, (**E**) the sub-cohort of ESMO-ESGO high risk patients, (**F**) the sub-cohort of ESMO-ESGO advanced/metastatic patients.

A 5-year PFS analysis conducted on the entire cohort revealed a significantly higher risk of disease progression in patients treated at MVCs than that for those treated at HVCs (*p* < 0.00057) (Figure 4A). A more stratified risk-based analysis demonstrated a significant impact of treatment center volume on PFS, particularly among patients classified in the high–intermediate- and high-risk groups (*p* = 0.0094). Conversely, no significant center-related effect on PFS was observed in patients within the low- and intermediate-risk groups or in those with advanced/metastatic disease (Figure 4B–D).

The impact of the volume of the center in these patient groups was also confirmed in a multivariate Cox model that included the main clinical variables. Supporting this finding, the analysis conducted on the entire cohort identified age (HR = 1.02, *p* = 0.007), risk group (high risk: HR = 4.04, *p* < 0.001, advanced/metastatic: HR = 11.7, *p* < 0.001), center (HVCs: HR = 0.68, *p* = 0.044), and adjuvant treatment (HR = 2.17, *p* < 0.001) as the main factors influencing PFS (Supplementary Figure S1A). Similarly, within the high–intermediate- and high-risk subgroup, in addition to confirming the worse prognosis of high-risk patients compared to high–intermediate ones (HR = 2.18, *p* = 0.003), both the treatment center (HVCs: HR = 0.60, *p* = 0.015) and adjuvant therapy (HR = 1.61, *p* = 0.048) remained significant predictors of PFS (Supplementary Figure S1B).

To explore whether the influence of the center extended to a broader subgroup of patients, we performed a PFS analysis including all patients in the intermediate-, intermediate–high-, and high-risk groups. Kaplan–Meier curves confirmed significant benefit of treatment at HVCs in prolonging PFS (*p* = 0.026) (Supplementary Figure S2A). However, in the multivariate analysis, the effect of the center showed a borderline statistical significance (*p* = 0.05), likely due to the strong impact of the risk class across such a heterogeneous patient population (Supplementary Figure S2B).

Moreover, recurrence analysis showed that patients treated in HVCs who experienced a recurrence compared with recurred patients treated in MVCs had a higher percentage of loco-regional relapses (34% (54/161) vs. 14% (10/74)), despite extra-abdominal presentation (34% (55/161) vs. 54% (40/74)) (Supplementary Figure S3).

A similar analysis was conducted to evaluate the effect of center volume on overall survival. Kaplan–Meier curves from the total cohort showed a significant difference in OS between women treated in MVCs and HVCs, with those treated in HVCs demonstrating a higher probability of survival over a 5-year follow-up period (*p* = 0.018) (Supplementary Figure S4A). However, in the multivariate analysis, the effect of center volume on OS was no longer statistically significant, likely due to the strong impact of age, ASA score, and ESMO-ESGO risk group (Supplementary Figure S4B). A more detailed stratified analysis, similar to that performed for PFS, conducted on low- and intermediate-risk, intermediate–high- and high-risk, and advanced/metastatic patients did not show a significant effect for center volume on OS.

## 4. Discussion

HVCs improved EC, reducing surgical morbidity and increasing both PFS and OS despite treating mostly patients at higher risk of recurrence. Most of the HVC patients were treated by LPS and received more-accurate surgical staging (more peritoneal biopsies, more lymph nodes resected, more positive lymph nodes harvested) than MVC patients, especially patients at high risk of recurrence. Patients managed at HVCs received adjuvant therapy less frequently than patients managed at MVCs, but when adjuvant therapy was received, chemotherapy was administered more frequently in patients managed at HVCs. These differences were especially significant in patients at high risk of recurrence. Patients at low risk of recurrence treated at HVCs relapsed more frequently than those treated at MVCs, but no difference in the number of recurrences was found in other recurrence risk classes, even though adjuvant therapy was administered less frequently in HVCs. In the multivariate Cox proportional hazards model, adjuvant therapy was associated with worse progression-free survival (PFS) (HR > 1), particularly within the total cohort. This finding was anticipated and likely reflects confounding by indication, as patients with higher-risk clinical features were more frequently selected for adjuvant treatment. Consequently, when the analysis was restricted to homogeneous risk classes, the negative impact of adjuvant therapy on PFS was substantially attenuated. Survival analysis revealed that patients at HVCs exhibited significantly improved PFS and OS. Specifically, multivariate analysis identified HVCs as an independent positive prognostic factor for PFS among intermediate–high- and high-risk subgroups. However, this protective effect did not extend to OS in the multivariate model. While HVCs remained a robust independent predictor of superior PFS, their impact was attenuated when accounting for heterogeneous recurrence risk groups—a trend that became even more pronounced in the OS analysis. Interestingly, HVC patients relapsed more frequently locally than MVC patients, who presented more extra-abdominal recurrences, particularly in high-risk patients. However, the interpretability of our findings may be constrained by the limited sample size within specific subgroups and the absence of comprehensive molecular data. This limitation is particularly relevant when evaluating distant recurrence patterns in non-endometrioid and p53-abnormal (p53-abn) enriched populations. Finally, although patients at higher risk of recurrence should be centralized to improve oncological outcomes, centralization may still positively reduce surgical morbidity in patients at low risk of recurrence or with advanced metastatic disease, where tumor biology appears to have a greater impact on prognosis, regardless of treatment center volume. Although scientific societies recommend centralizing EC patient treatments, the literature is controversial. Dutch [23] and American [11] registry studies showed no impact or only a modest impact of volume on survival. Instead, other registry studies showed that hospital volume significantly increased survival outcomes [24,25] and was associated with better efficiency and cost-effective treatments [12]. Other studies found that centralization would not be useful to improve EC survival [13,23]; only high-risk cases should be selected and referred to HVCs [10,13]. On the contrary, most recent studies showed that HVCs improved OS only of stage I [26] and type 1 EC patients [15]. Therefore, some authors have suggested some algorithms to safely select patients to be centralized in HVCs [9,27]. Centralization and treatment by gynecologic oncologists have been associated with improved quality of care, with lower postoperative morbidity and adequate surgical staging. Consequently, improved patient selection for adjuvant treatment, particularly chemotherapy, has been observed, especially in high-risk patients [28,29,30]. Moreover, treatment in HVCs may reduce the survival disparity between black and white women with EC [14]. However, the centralization of care for EC is not without criticism. While it is widely associated with better surgical outcomes and adherence to guidelines, it presents several logistical and socio-ethical challenges. Centralization inevitably leads to longer travel distances for patients living in rural or peripheral areas. This can be physically exhausting for patients who are often elderly or have comorbidities; travel costs, lost wages for caregivers, and the logistical difficulty of arranging transportation can create significant barriers, potentially exacerbating existing socioeconomic inequalities in healthcare access. Centralization may favor patients living in urban centers near tertiary hospitals, while those in peripheral regions may suffer from delayed diagnosis or suboptimal initial management. If the number of designated reference centers is too low, these institutions may become overwhelmed. This can lead to increased waiting times for surgery or consultations, which may negatively affect oncological outcomes. Several papers investigated the impact of the delay of surgery on EC oncological outcomes [31,32,33,34,35,36,37,38]. The experience of the COVID-19 pandemic has demonstrated how diagnostic delay can lead to a delayed diagnosis with a consequent increase in the stage and aggressiveness of EC at diagnosis [39]. Another study reported that delaying surgery by more than eight weeks had no impact on patient mortality but increased the need for adjuvant pelvic radiation therapy and worsened recurrence rates [31]. A previous large population-based study demonstrated that patients experiencing wait times exceeding 12 weeks exhibited significantly poorer survival outcomes than those treated within the 2.1–12-week interval [32]. However, particularly in low-risk patient populations, treatment delays showed little or no correlation with stage progression, likely due to a greater interaction with tumor biology [33,34]. On the contrary, a previous paper showed that treatment delays were identified as a significant risk factor for mortality exclusively in low-risk EC, likely reflecting restricted access to specialized care. However, the authors concluded that referring to an experienced surgical team and thorough preoperative optimization should be prioritized over expedited surgery [35]. A new paper reported that surgical delays beyond four weeks were associated with reduced PFS and increased recurrence [36]. Recent reviews showed that the most common cut-off for the time to surgery interval was six weeks, and for the time to adjuvant treatment, nine weeks. Only 24–74% of EC patients were treated according to this indication [37,38]. These reviews suggested performing surgery between two and eight weeks from diagnosis [37,38]. While the centralization of EC patients within HVCs may result in surgical delays and a dilution of clinical proficiency among gynecologists in LVCs—potentially compromising their readiness to manage unexpected complications—this organizational model remains preferable and should be vigorously supported. However, HVCs must extend their role beyond the surgical procedure to lead multidisciplinary management, ranging from the selection of adjuvant therapies to the coordination of follow-up. Simultaneously, they must provide consistent support and continuous training to LVCs to ensure their operational resilience. This study presents several limitations due to its retrospective design and long observation period; furthermore, the lack of data may have affected the quality of the study. Some therapeutic choices, such as performing lymphadenectomy or adjuvant therapy in patients at low risk of recurrence, or conversely, omitting them in patients at high risk of recurrence, may not always be clearly justified. Furthermore, the lack of molecular data may render the study obsolete. In the absence of molecular data for the entire study population, it can be reasonably assumed that this limitation is evenly distributed across groups and therefore does not significantly impact the overall results. Notably, even when patients are considered as a non-stratified cohort, the volume of cases treated by the institution appears to impact prognosis; centers with high case volumes tend to achieve better clinical outcomes. On the other hand, the multicenter design and large sample provide valuable insights into the evolution of endometrial cancer treatment over recent decades, reflecting real-world clinical practice. Since the study involved a region with a high rate of centralization (absence of low-volume centers), the data should be more reliable. Higher surgical volumes were expected to improve the quality of surgery and overall care through standardization. Unfortunately, the low number of advanced/metastatic patients included in the study population may have prevented us from confirming that centralization also improves the prognosis of these patients, as recently reported [40]. Despite significant advances in therapeutic strategies, oncological outcomes for patients with advanced EC remain suboptimal. Recently, while the role of neoadjuvant chemotherapy is still debated, it could represent a key treatment modality, particularly for cases characterized by unresectable primary disease. Furthermore, the integration of specific histological subtypes and molecular profiling is essential to refine patient selection and potentially improve survival outcomes in this high-risk population [41,42,43,44,45].

## 5. Conclusions

Centralization of EC patients should be promoted because it is associated with lower perioperative morbidity and better quality of care. Patients treated at HVCs, especially those at high risk of recurrence, underwent more-frequent LPSs and achieved more-accurate surgical staging (higher counts of biopsies, resected nodes, and positive nodes) than those at MVCs. Patients treated at HVCs showed longer PFS, in particular in intermediate–high- and high-risk patients. All EC patients could be centralized to receive higher quality treatment to improve recovery from surgery and improve oncologic outcome, particularly for patients with more-aggressive cancers. New studies are needed to establish a globally uniform minimum annual case volume of treated EC patients required to designate a hospital as a referral center.

## Figures and Tables

**Table 1:** Characteristics of EC patients treated in medium-volume centers (MVCs) and high-volume centers (HVCs).

	MVCs (N = 971)	HVCs (N = 1431)	Total (N = 2402)	*p*-Value
**Age (years)**				0.003
Mean (SD)	66.4 (10.9)	65.0 (10.8)	65.6 (10.9)	
N-Miss	7	2	9	
**BMI**				0.255
Normal weight	214 (23.6%)	317 (26.8%)	531 (25.4%)	
Overweight	298 (32.8%)	374 (31.6%)	672 (32.1%)	
Obese	396 (43.6%)	493 (41.6%)	889 (42.5%)	
N-Miss	63	247	310	
**ASA score**				<0.001
I	60 (6.6%)	99 (7.4%)	159 (7.1%)	
II	457 (50.1%)	768 (57.8%)	1225 (54.7%)	
III	386 (42.3%)	447 (33.6%)	833 (37.2%)	
IV	9 (1.0%)	15 (1.1%)	24 (1.1%)	
N-Miss	59	102	161	
**Hypertension**				0.016
No	406 (44.2%)	665 (49.4%)	1071 (47.3%)	
Yes	513 (55.8%)	682 (50.6%)	1195 (52.7%)	
N-Miss	52	84	136	
**Diabetes**				0.688
No	769 (83.7%)	1108 (82.9%)	1877 (83.2%)	
Yes	150 (16.3%)	228 (17.1%)	378 (16.8%)	
N-Miss	52	95	147	
**Comorbidities**				<0.001
No	418 (45.5%)	579 (58.8%)	997 (52.4%)	
Yes	500 (54.5%)	406 (41.2%)	906 (47.6%)	
N-Miss	53	446	499	
**Surgical approach**				<0.001
Laparotomy	493 (50.8%)	544 (38.3%)	1037 (43.4%)	
Laparoscopy	458 (47.2%)	825 (58.1%)	1283 (53.6%)	
Vaginal hysterectomy	20 (2.1%)	52 (3.7%)	72 (3.0%)	
N-Miss	0	10	10	
**Hemoglobin variation (g/dL)**				<0.001
Mean (SD)	−1.8 (1.1)	−1.6 (1.2)	−1.7 (1.1)	
N-Miss	81	217	298	
**Surgical time (Minutes)**				<0.001
Mean (SD)	177.1 (59.5)	145.2 (69.2)	158.2 (67.3)	
N-Miss	76	121	197	
**Days of hospitalization**				<0.001
Mean (SD)	6.5 (4.9)	5.7 (3.5)	6.0 (4.2)	
N-Miss	55	126	181	
**Peritoneal biopsies**				<0.001
No	839 (86.5%)	1019 (72.6%)	1858 (78.3%)	
Yes	131 (13.5%)	385 (27.4%)	516 (21.7%)	
N-Miss	1	27	28	
**Pelvic lymphadenectomy**				0.134
No	489 (50.5%)	676 (47.3%)	1165 (48.6%)	
Yes	479 (49.5%)	752 (52.7%)	1231 (51.4%)	
N-Miss	3	3	6	
**Lumbo-aortic lymphadenectomy**				0.729
No	823 (85.0%)	1206 (84.5%)	2029 (84.7%)	
Yes	145 (15.0%)	222 (15.5%)	367 (15.3%)	
N-Miss	3	3	6	
**Total number of excised lymph nodes**				<0.001
Mean (SD)	19.0 (13.4)	21.5 (11.5)	20.5 (12.4)	
N-Miss	31	71	102	
**Percentage of positive lymph nodes**				0.024
Mean % (SD)	4.2 (13.8)	2.4 (9.9)	3.5 (12.5)	
N-Miss	85	89	174	
**FIGO stage**				0.161
I	784 (81.4%)	1126 (80.7%)	1910 (81.0%)	
II	38 (3.9%)	68 (4.9%)	106 (4.5%)	
III	129 (13.4%)	169 (12.1%)	298 (12.6%)	
IV	12 (1.2%)	32 (2.3%)	44 (1.9%)	
N-Miss	8	36	44	
**Grade (Endometrioid EC only)**				0.018
G1-G2	715 (81.5%)	981 (85.5%)	1696 (83.8%)	
G3	162 (18.5%)	166 (14.5%)	328 (16.2%)	
N-Miss	4	1	5	
**Histology**				<0.001
Endometrioid	881 (90.9%)	1148 (80.5%)	2029 (84.7%)	
Other histotypes	88 (9.1%)	278 (19.5%)	366 (15.3%)	
N-Miss	2	5	7	
**LVSI**				0.015
No	704 (79.2%)	914 (74.6%)	1618 (76.5%)	
Yes	185 (20.8%)	311 (25.4%)	496 (23.5%)	
N-Miss	82	206	288	
**ESMO-ESGO risk**				<0.001
Low	380 (40.6%)	532 (41.1%)	912 (40.9%)	
Intermediate	147 (15.7%)	132 (10.2%)	279 (12.5%)	
Intermediate/high	119 (12.7%)	171 (13.2%)	290 (13.0%)	
High	279 (29.8%)	428 (33.1%)	707 (31.7%)	
Advanced/metastatic	12 (1.3%)	32 (2.5%)	44 (2.0%)	
N-Miss	34	136	170	
**Adjuvant treatment**				<0.001
No	485 (49.9%)	856 (59.8%)	1341 (55.8%)	
Yes	486 (50.1%)	575 (40.2%)	1061 (44.2%)	
N-Miss	0	0	0	
**Type of adjuvant treatment**				<0.001
Brachytherapy	167 (34.5%)	160 (28.2%)	327 (31.1%)	
Chemotherapy	138 (28.5%)	243 (42.9%)	381 (36.3%)	
Radiotherapy	179 (37.0%)	164 (28.9%)	343 (32.6%)	
N-Miss	2	8	10	

N-Miss: missing data, BMI: body mass index, ASA: American Society of Anesthesiologists, FIGO: International Federation of Gynaecology and Obstetrics, LVSI: lymphovascular space invasion, ESMO: European Society for Medical Oncology, ESGO: European Society of Gynaecological Oncology, SD: standard deviation. The bold text identifies the variable under analysis.

## Data Availability

Data is available from the authors upon reasonable request.

## Supplementary Materials

The following supporting information can be downloaded at: https://www.mdpi.com/article/10.3390/cancers18071050/s1, Figure S1: Forest plot summarizing the multivariate Cox regression results for progression-free survival, incorporating center volume and main clinical covariates in the overall cohort (A) and in the sub-cohort of ESMO-ESGO intermediate–high- and high-risk patients (B); Figure S2: (A) Kaplan–Meier curves illustrating the effect of center volume on progression-free survival in the sub-cohort of ESMO-ESGO intermediate, intermediate–high- and high-risk patients. (B) Forest plot summarizing the multivariate Cox regression results for progression-free survival, incorporating center volume and main clinical covariates in the sub-cohort of ESMO-ESGO intermediate, intermediate–high- and high-risk patients. Figure S3: Pie charts showing the distribution of recurrence patterns (loco-regional, abdominal, and extra-abdominal) in patients treated at medium-volume centers and high-volume centers. Figure S4: (A) Kaplan–Meier curves illustrating the effect of center volume on overall survival in the total cohort. (B) Forest plot summarizing the multivariate Cox regression results for overall survival, incorporating center volume and main clinical covariates in total cohort of EC patients.
