# Supplementary material for: Impact of Facility Volume on Therapy and Survival for Endometrial Cancer: A Retrospective Multicenter Study
Source: Cancers (Basel). 2026 Mar 24;18(7):1050. doi: 10.3390/cancers18071050 (PMC13072166; doi:10.3390/cancers18071050)
Supplement: Supplementary file 1 [file cancers-18-01050-s001.zip › cancers-4121536-supplementary.pdf]

cancers

MDPI

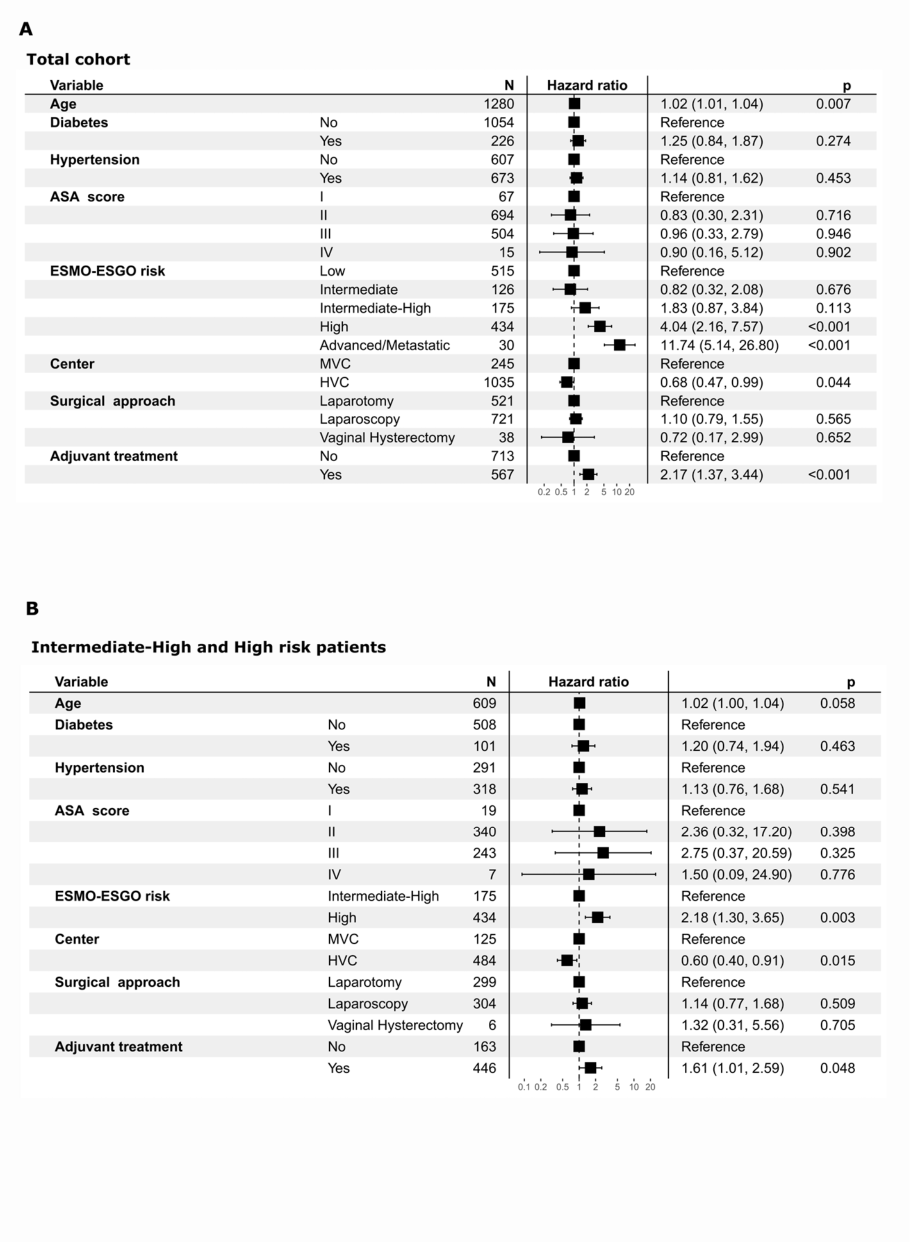

Supplementary Figure S1. Forest plot summarizing the multivariate Cox regression results for progression-free survival, incorporating center volume and main clinical covariates in the overall cohort (A) and in the sub-cohort of ESMO-ESGO intermediate-high- and high-risk patients (B).

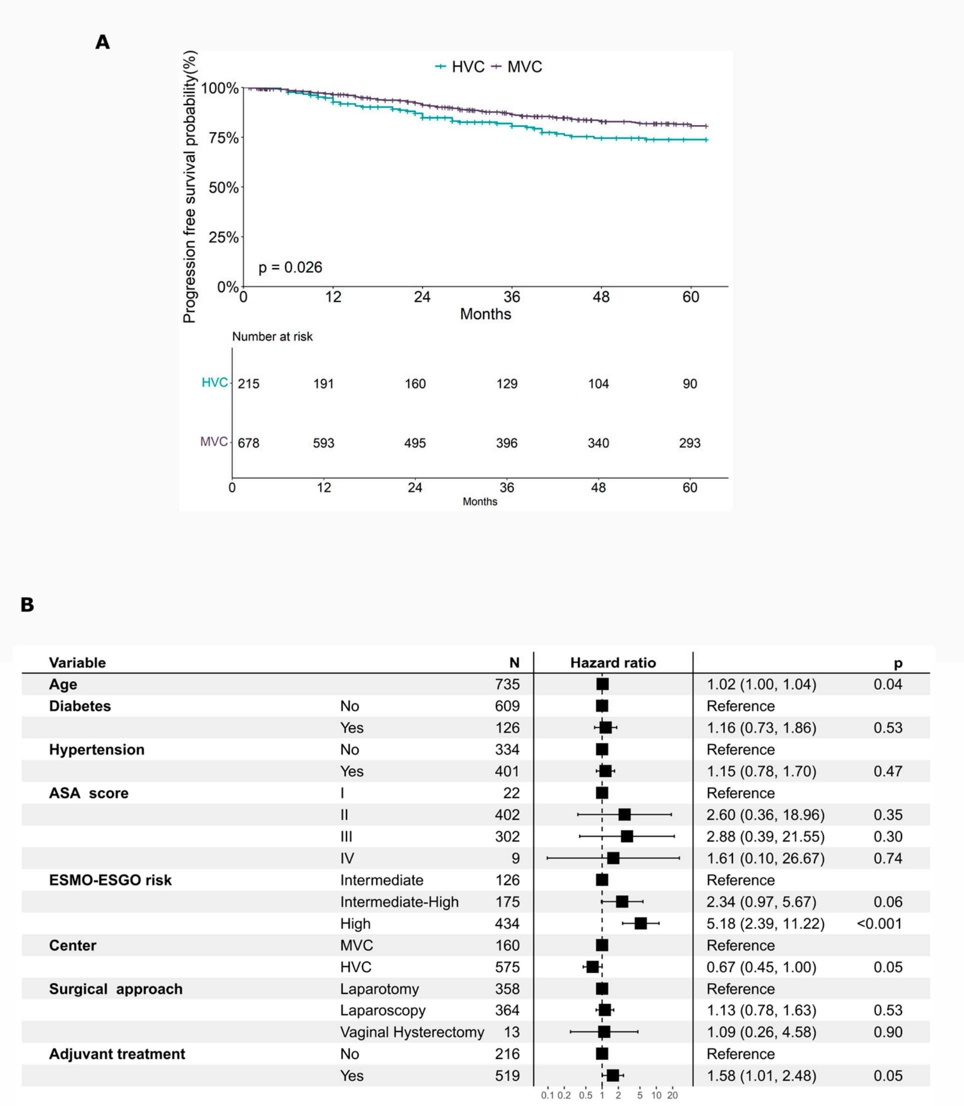

Supplementary Figure S2. A) Kaplan–Meier curves illustrating the effect of center volume on progression-free survival in the sub-cohort of ESMO-ESGO intermediate, intermediate-high- and high-risk patients. B) Forest plot summarizing the multivariate Cox regression results for progression-free survival, incorporating center volume and main clinical covariates in the sub-cohort of ESMO-ESGO intermediate, intermediate-high and high-risk patients.

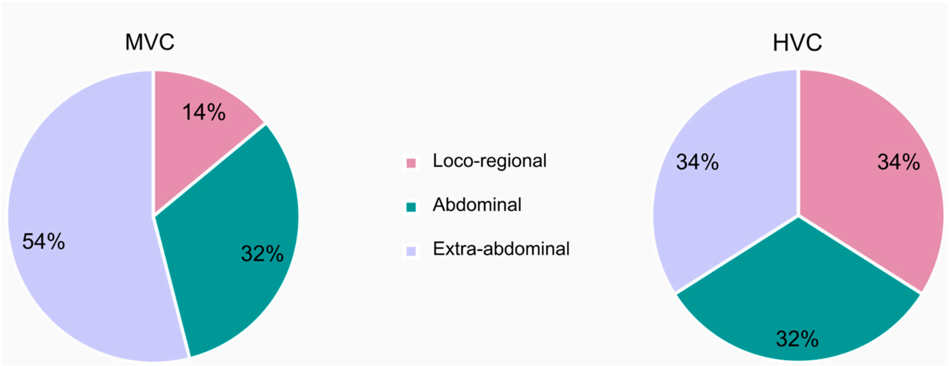

Supplementary Figure S3. Pie charts showing the distribution of recurrence patterns (loco-regional, abdominal, and extra-abdominal) in patients treated at medium volume centers and high volume centers.

A

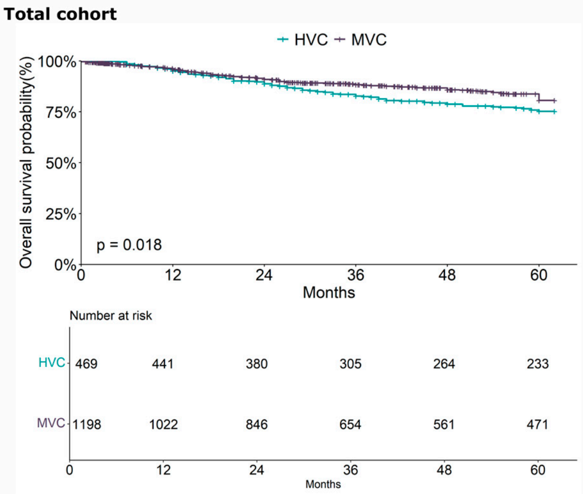

B

Total cohort

Variable		N	HR (95% CI)	p
**Age**		1292	1.06 (1.04, 1.07)	<0.001
**Diabetes**	No	1066	Reference	
	Yes	226	1.21 (0.86, 1.69)	0.28
**Hypertension**	No	614	Reference	
	Yes	678	1.06 (0.78, 1.43)	0.72
**ASA score**	I	74	Reference	
	II	701	1.43 (0.52, 3.94)	0.49
	III	500	2.10 (0.76, 5.87)	0.15
	IV	17	4.33 (1.19, 15.78)	0.03
**ESMO-ESGO risk**	Low	500	Reference	
	Intermediate	152	1.41 (0.81, 2.46)	0.23
	Intermediate-High	151	1.57 (0.82, 3.01)	0.18
	High	458	3.34 (2.16, 5.17)	<0.001
	Advanced/Metastatic	31	16.67 (8.89, 31.27)	<0.001
**Center**	MVC	395	Reference	
	HVC	897	1.04 (0.77, 1.41)	0.78
**Surgical approach**	Laparotomy	652	Reference	
	Laparoscopy	606	0.75 (0.55, 1.02)	0.07
	Vaginal Hysterectomy	34	1.32 (0.68, 2.55)	0.41
**Adjuvant treatment**	No	707	Reference	
	Yes	585	1.10 (0.79, 1.53)	0.57

Hazard ratio axis: 1 2 5 10 20

Supplementary Figure S4. A) Kaplan–Meier curves illustrating the effect of center volume on overall survival in the total cohort. B) Forest plot summarizing the multivariate Cox regression results for overall survival, incorporating center volume and main clinical covariates in total cohort of EC patients.
